# Smartphone-Based Contingency Management for Patients Who Use Methamphetamine: Qualitative Analysis of Patient and Clinician Perspectives

**DOI:** 10.2196/80808

**Published:** 2026-02-19

**Authors:** Yanni M Chang, Adam C Ketron, Mark H Duncan, Matthew D Iles-Shih, Andrew J Saxon, Kevin A Hallgren

**Affiliations:** 1 Department of Psychiatry & Behavioral Sciences University of Washington Seattle, WA United States; 2 Center of Excellence in Substance Addiction Treatment and Education VA Puget Sound Health Care System Seattle United States

**Keywords:** contingency management, DynamiCare, methamphetamine use disorder, mHealth, mobile health, motivational incentives, primary care, qualitative study, stimulant use disorder

## Abstract

**Background:**

Methamphetamine use disorder is a growing public health crisis with limited access to effective treatment. Contingency management (CM) has demonstrated efficacy for stimulant use disorders, but is typically delivered in person. Smartphone-based CM may overcome barriers such as limited access, but its effectiveness and real-world application remain understudied. This study explores patient and clinician experiences with a fully remote, smartphone-based CM intervention for methamphetamine use.

**Objective:**

This exploratory, descriptive qualitative study analyzes interviews with patients and clinicians involved in a previously published single-arm trial in which smartphone-based CM was offered to individuals using methamphetamine through primary care or specialty addiction treatment clinics within a large health system. The study aims to identify and describe key facilitators, barriers, and perspectives related to engagement of both groups with the intervention, providing actionable insights to inform optimization and implementation of digital CM in health care settings.

**Methods:**

We conducted a qualitative analysis of semistructured interviews with 14 patients and 14 clinicians from a prior pilot study of a fully remote, smartphone-based CM intervention for methamphetamine use. Interviews were analyzed using grounded theory in a 5-step process: transcript review, codebook development, coding, thematic reduction, and generation of overarching themes. The analysis focused on a priori themes related to facilitators, barriers, and suggestions for improvement.

**Results:**

Patients and clinicians identified many benefits, viewing the program as valuable for individuals using methamphetamine. Patients appreciated the flexibility, accessibility, and motivational incentives. Clinicians saw CM as a low-risk, evidence-based strategy that could enhance engagement, especially among patients less responsive to traditional approaches. Common challenges included technological issues such as problems with video-based testing, app navigation, and internet access. Patients had mixed views about educational modules and described difficulty with correct substance test procedures and a lack of human connection. Clinicians expressed concerns for patients with significant psychosocial instability. Differences emerged in the types of concerns raised: patients focused on day-to-day engagement, while clinicians emphasized broader themes of equity, sustainability, and a preference for models rewarding improvement even without full abstinence.

**Conclusions:**

Smartphone-based CM shows promise for addressing methamphetamine use disorder, especially in settings lacking traditional treatment access. However, optimizing implementation requires addressing challenges related to technology, accessibility, and equity. Recommendations include integrating CM with clinical infrastructure, expanding rewardable behaviors beyond abstinence, enhancing user experience, and improving technological access. Future research should explore flexible models that incorporate broader recovery goals and strengthen both technical and human support.

## Introduction

Methamphetamine use disorder (MUD) is an escalating public health issue in the United States, where methamphetamine-involved drug overdose deaths have risen more than 5-fold from 2015-2022 [1]. To combat the significant impact of MUD, more efforts are needed to increase the availability and accessibility of treatments for methamphetamine use.

Currently, the MUD treatment with the strongest evidence base is contingency management (CM), a behavioral intervention in which motivational incentives (eg, financial rewards) are administered to patients when they provide objective evidence of a target behavior (eg, objective evidence of recent methamphetamine abstinence as verified by a substance toxicology test) [2,3]. Recent guidelines jointly issued by the American Society of Addiction Medicine and the American Academy of Addiction Psychiatry identify CM as the intervention with the best evidence of effectiveness for the treatment of stimulant use disorders and state that CM should represent the current standard of care for treatment of stimulant use disorders [4]. However, despite the efficacy of CM, there have been major challenges to implementing it in health care settings, and it is almost never available in more general medical settings like primary care. Limited access to CM is due to multiple implementation barriers, including intervention cost, regulatory policies, stakeholder buy-in, and clinical staffing and resource barriers related to collecting urine/saliva samples and delivering rewards several times per week [5,6].

While digital CM approaches have begun to show feasibility and efficacy in broad substance use disorder (SUD) and opioid use disorder (OUD) populations, very few studies have examined these methods in the specific context of MUD. Given the unique features of MUD (eg, lack of approved pharmacotherapies), this represents an important gap in the evidence base [4].

Smartphone-based CM interventions may also help address many of the structural and logistical barriers that have limited access to traditional, clinic-based CM. By allowing patients to complete substance tests and receive incentives remotely, these digital platforms can reduce the burden of frequent in-person visits, lessen demands on clinic staff, and facilitate implementation in primary care and other settings without specialized CM infrastructure. The use of mobile technology may therefore extend the reach of this evidence-based intervention to individuals and health care systems previously unable to offer or access CM.

Several studies have demonstrated that CM can be offered digitally using mobile devices [7,8]. For example, using smartphones or other devices, patients can complete the essential components of CM interventions by videorecording themselves taking saliva-based substance toxicology tests, which program staff review to verify methamphetamine abstinence and disburse financial incentives through reloadable debit cards [9-11]. This approach could help make CM more accessible by allowing patients to engage with CM from any location (ie, not needing to come into the clinic to complete substance tests or receive financial incentives) and potentially making CM available in clinical settings that otherwise would not have the capacity to maintain a CM program.

During the COVID-19 pandemic, which posed significant barriers to in-person clinical services, our team studied the feasibility and usability of a commercially available mobile health (mHealth) CM intervention as part of an effort to increase the availability of CM in primary care and addiction treatment clinics [12]. The pilot study found that smartphone-based CM was feasible to deliver to patients who used methamphetamine and who were receiving treatment within clinical settings that lacked CM programs. However, despite patients’ satisfaction with the intervention, there was limited engagement with core intervention components (eg, participants completed 35% of substance tests requested) and limited rates of verified methamphetamine abstinence (eg, verified methamphetamine abstinence on 31% of substance tests completed).

Considering these findings, there is a need to better understand factors that could influence engagement with and the success of smartphone-based CM interventions. This study was designed as a descriptive, exploratory investigation aimed at identifying potential barriers, facilitators, and ideas for improving the mHealth-based CM intervention offered in the pilot study. To achieve this, we conducted qualitative analyses of interviews with both patients who received the intervention and clinicians involved in offering it to them, capturing multiple perspectives on its implementation and usability. We hope that these findings, in turn, could be used to provide actionable insights to help guide the future development and implementation of mHealth-based CM interventions.

## Methods

### Participants and Procedures

As previously described [12], patients receiving treatment from 7 primary care clinics or 1 specialty SUD clinic were invited to participate in a single-arm pilot study in which they would receive access to the mHealth CM intervention for up to 3 months. Patient eligibility criteria included (1) receiving care from a participating clinic, (2) self-reported methamphetamine use for at least 5 out of the past 30 days, (3) a self-reported goal of reducing or abstaining from methamphetamine use, (4) age 18 years or older, and (5) ability to read and communicate in English per self-report. Patients without smartphones who met eligibility criteria could receive a smartphone to use through the study. The study recruitment period was from September 2021 to July 2022. Clinic staff were provided with information about the study and flyers that they could distribute to patients they thought might benefit from CM; patients could also self-refer by calling the phone number listed on the flyers that had been posted in public clinical spaces.

### Ethical Considerations

The study procedures were approved by the University of Washington Institutional Review Board (STUDY00013066). All research activities were conducted in accordance with institutional and national ethical standards, as well as the principles of the Declaration of Helsinki. Participants provided informed consent prior to participation, and all data were collected and stored to maintain privacy and confidentiality. Research procedures were completed remotely via telephone, videoconference, SMS text message, and the smartphone app. Participants were not financially compensated beyond the incentives provided as part of the intervention itself.

### Intervention Description

The DynamiCare Health intervention (Boston, Massachusetts) was adapted and used as the intervention for the separately published pilot study. While previously evaluated for nicotine, alcohol, and OUDs [9-11,13-16], this study was the first to our awareness to apply the DynamiCare Health intervention specifically to methamphetamine use. The intervention’s components were tailored to suit the study’s needs, including intervention duration, reward structures, and specific cognitive behavioral therapy (CBT) modules. Onboarding procedures were modified based on clinician input obtained prior to the study, with the goal of minimizing workflow disruptions.

After enrolling in the study, participants entered a “welcome phase” in which they were required to download the smartphone app, receive testing supplies and a reloadable debit card by mail, and complete 2 instructional video calls in which they were provided opportunities to become familiar with the platform. During the welcome phase, participants also needed to perform 2 “practice” substance tests within 6 hours of being prompted by the app to verify their ability to complete this core intervention component. DynamiCare staff reviewed these recordings to verify participant identity, proper test administration, and result visibility. Participants earned US $10 per video call and US $5 per practice test.

Upon completing the welcome phase, participants received access to the 12-week intervention. The app randomly prompted saliva-based substance testing twice weekly, offering an US $8.42 incentive for methamphetamine and amphetamine abstinence (rewards were not contingent on abstinence from other substances). Reward values increased, and testing frequency decreased with consecutive negative tests. Participants received OralTox Oral Fluid Drug Tests (Premier Biotech) by mail, which tested for multiple substances and included separate panels for methamphetamine (cutoff of 50 ng/mL) and other amphetamines (cutoff of 50 ng/mL; only methamphetamine and amphetamine results were factored into the financial incentive structure).

The intervention included access to 35 brief, self-paced CBT modules, with participants earning US $1 per completed module. Up to 9 surveys were prompted through the app, rewarding US $1 or US $2 per completion. Participants had access to a CM guide with a lived recovery experience who provided encouragement and support with completing the intervention components. Weekly meetings with the CM guide initially were not incentivized, then later incentivized to boost engagement (US $20 for the first meeting and US $10 for each meeting thereafter). Participants could earn up to US $45 more for completing research assessments at baseline, 6 weeks, and 12 weeks. Total potential earnings varied based on enrollment timing. Participants completing the welcome phase before March 2022 (before CM guide meetings were incentivized) could earn up to US $325, while those enrolling later could earn up to US $465, including research assessment compensation. Referring clinicians were not actively involved in the intervention but could access a web-based dashboard to monitor patient progress and received monthly secure message updates summarizing their patients’ participation.

### Qualitative Data Collection

Patients who completed the welcome phase were invited to participate in semistructured interviews via telephone halfway through the intervention period (ie, after the sixth week of the 12-week intervention). Clinicians who referred patients to the intervention or who were identified as providing SUD or other behavioral health care services to study participants were identified by reviewing participants’ electronic health records, and these clinicians were also invited to participate in semistructured interviews via Zoom (Zoom Communications) shortly after the completion of the trial.

Patient interview questions solicited information about both positive aspects (facilitators) and negative aspects (barriers) of the intervention—what they found most or least helpful, what made the intervention easy or difficult to use, how participation with the intervention affected their substance use—as well as suggestions on how to improve the intervention and how to advertise the intervention to other patients. Clinician interview questions also focused on facilitators and barriers related to their experience with the intervention—how it was helpful to their patients, how it fit into their clinical workflow, considerations about implementing such an intervention in their clinics—and similarly asked for suggestions on how the intervention might be improved both in its current form and if the intervention were to be implemented in their clinical setting in a longer-term manner (ie, beyond the scope of this small pilot study). Patient interviews were transcribed near-verbatim in real time with occasional summarization; clinician interviews were audio recorded and transcribed verbatim.

### Analytic Plan

Two members of the research team (YMC and ACK, both psychiatry residents at the time of coding) conducted a qualitative analysis of the transcribed interviews with guidance from a third team member (KAH, academic psychologist). Analyses proceeded according to a 5-step plan (outlined below) based on grounded theory, which aims to generate themes grounded in the data, as described by Felner and Henderson [17]. This analytic framework was selected because it reflects a rigorous, iterative approach to deriving themes from transcripts that still fit the timeline available to the investigative team.

First, all transcripts were reviewed, and analytic memos (informal analytic notes about the data and theoretical connections) were applied to specific segments or excerpts to better understand the range of participants’ thoughts. These memos were then compiled, and a codebook of inductive codes grounded in the data was developed, with each code fitting under 3 a priori themes aligning with the focus of topics addressed in each interview: facilitators or benefits of the intervention, barriers or drawbacks, and ideas for improving it. Codes were then applied to discrete segments of the text. Next, all coded text segments associated with a single code were grouped and reduced by writing narrative summaries for each code. Finally, themes were generated by systematic analytic engagement with the data, including discussions among the investigative team looking for patterns that emerged from the data. Concurrently, thematic networks were developed to visually connect ideas between themes and codes.

This multistep process was completed iteratively; that is, earlier steps were repeated and clarified to achieve a more granular understanding of the data as the researchers’ experience with the data grew and became more refined. Dedoose (Los Angeles, California), a software used for codifying and analyzing qualitative and mixed methods research, was used to organize the approach, to house the codebooks, and to facilitate coding and analysis of the transcripts.

The authors selected 6 transcripts to be coded independently by both coders. These transcripts were then reviewed in consultation with the senior researcher to identify areas of concordance and divergence in code application. Discussions from this review were used to refine code definitions, clarify boundaries between similar codes, and ensure shared understanding of analytic concepts. Once an agreement in interpretive approach was established, the remaining transcripts were coded by a single coder. Because the analytic framework used was grounded in interpretive, inductive qualitative methodology and focused on latent meaning rather than surface-level semantic features, formal quantitative intercoder agreement statistics were not calculated, as such measures are not always appropriate or meaningful for interpretive qualitative approaches and may conflict with underlying epistemological assumptions [18]. Throughout the process, reflexive discussion among the coding team was used to promote analytic consistency, consider alternative interpretations, and enhance the credibility of the final themes.

## Results

### Description of Patient Sample

#### Overview

A total of 28 patients consented to enroll in the pilot trial, of whom 15 completed an initial welcome phase and received access to the intervention. Of these, 14 participants completed semistructured interviews at the midtreatment research assessment and were included in this study. Characteristics of these participants are shown in Table 1. In brief, patients were predominantly male, half were White or Caucasian, and the other half were from other racial and ethnic groups. Just under half were members of a sexual minority group. Most reported using methamphetamine on 15 or more days out of the past 30, and most expressed a goal of abstinence from methamphetamine use (as opposed to nonabstinent reduction in use). Half also received medications for OUD, most had another cooccurring nonamphetamine SUD documented in the electronic health record, and all had a cooccurring mental health disorder. Housing insecurity, financial insecurity, unemployment, and a lack of transportation were common. Themes identified from analyses of patient interviews are discussed in the sections below and are also summarized in Table 2, which includes a summary of each theme, example quotes, and the number of patients whose transcripts had any codes related to each identified theme.

**Table 1:** Characteristics of patient participants who completed qualitative interviews (n=14).

Characteristics		Value, n (%)
**Age (years)**		
	18-29	4 (29)
	30-45	5 (36)
	46-64	5 (36)
**Sex**		
	Female	4 (29)
	Male	10 (71)
**Race**		
	Black or African American	3 (21)
	Hispanic, Latinx, or Spanish origin	1 (7)
	Middle Eastern or North African	1 (7)
	White or Caucasian	7 (50)
	More than 1 race	2 (14)
**Member of a sexual minority group**		
	Yes	6 (43)
	No	8 (57)
**Past 30-day methamphetamine use**		
	5-9 days	1 (7)
	10-14 days	0 (0)
	15-20 days	7 (50)
	21-30 days	6 (43)
**Methamphetamine use goal**		
	Abstinence	10 (71)
	Nonabstinent reduction	4 (29)
Prescribed medications for opioid use disorder		7 (50)
Cooccurring nonamphetamine SUD^a^ (per EHR^b^)		11 (79)
Cooccurring mental health disorder (per EHR)		14 (100)
**Benefits**		
	Medicaid	11 (85)
	Disability	3 (23)
	Social Security	4 (31)
	Welfare	2 (15)
	Supplemental Nutrition Assistance Program	9 (69)
Unhoused or in temporary or transitional housing (n=13)^c^		1 (8)
Housing insecurity (n=12)^c^		4 (33)
Employed (n=11)^c^		2 (18)
Lack of transportation (n=13)^c^		7 (54)
Financial insecurity (n=13)^c,d^		7 (54)
Past-year jail or criminal legal involvement (n=13)^c^		2 (15)

^a^SUD: substance use disorder.

^b^EHR: electronic health record.

^c^There was 1 participant who did not provide information about housing status, transportation, financial insecurity, or past-year jail or criminal legal involvement; 2 participants who did not provide information about housing insecurity; and 3 participants who did not provide information about employment.

^d^Financial insecurity was defined by the self-reported inability to pay for at least 2 of the following things when needed in the past year: food, clothing, utilities, childcare, medicine/health care, phone, or other.

**Table 2:** Summary of themes identified by patients.

Theme		Summary	Example quotes	Patients, n	
**Facilitators (patients)**					
	Testing was helpful	Substance testing was seen as helpful for several reasons, including the ability to see test results in real time, the randomness of testing, the good feeling associated with passing a drug test, feeling encouraged to complete a drug test even after using substances, the flexible window for testing, the ability to show family that the patient has been compliant, and the ability to see what substances are present.	“I do like the structure of having random saliva tests, because that has motivated me. Like when I was about to relapse, I was like ‘I don’t want to not get paid for that and I don’t want to risk having a nonpositive test.”	8	
	Modules were helpful	Educational modules were seen as helpful for at least 2 reasons, including learning from the modules and enjoying completing the modules.	“I really enjoy reading the CBT modules and it’s nice and pleasant way of refreshing my head what’s wrong, what’s right, how to deal with things without really stressing out over things that aren’t in my control.”	6	
	Incentives were helpful	Patients expressed positive opinions about financial incentives, including having a reason to want to be in the program, benefit to daily life, the value of the financial incentive being enough, efficient delivery of incentives, and limits on what the card can be used to purchase.	“I liked the fact that it was a reward, an incentive with money. I like money quite a bit, just like everyone else does. And also just to see if that was enough to make me quit, so I was interested in trying it out.” “I think [the incentive values] were completely fair with today’s prices. I think it’s pretty spot on. It’s well done, calculated.”	11	
	Intervention fits into the landscape of other treatment services	Patients felt that the intervention complemented their overall treatment, including that it was a treatment option where there has otherwise been a lack of treatment options, was a more palatable alternative to rehabilitation, enhanced engagement with other mental health treatments, and did not interfere with or detract from other treatments.	“When you’re in my position, signing up for rehab seems like climbing Mount Everest times ten. And with DynamiCare it’s just the perfect amount of help for someone like me in my position.”	12	
	Technology / remote delivery	Patients appreciated technological aspects of the intervention, including not needing to travel to the clinic to participate in the intervention; helpful reminders; and a user-friendly interface.	“I thought it was very convenient, it comes in handy ... I thought it was great. It’s not like you needed to catch a bus to meet with you all, everything was done online.”	12	
	Accountability, helpfulness, and overall benefit of the intervention	Patients found the overall intervention helpful in several ways, including reducing substance use and promoting harm reduction, providing accountability, noticing more benefits with more time spent in the intervention, wanting to do or be “better,” meeting or exceeding patient expectations, and finding all parts of the intervention helpful.	“It’s a great program, it’s helped me stay sober and helped me in times of cravings and emotional vulnerability.” “Overall, I would say that it’s set up in a nice way. I can’t see anything I would add to benefit the program. It’s easy enough, understandable enough, it’s good.”	14	
	Human connection	Patients expressed a sense of human connection despite receiving care remotely, including that intervention staff were helpful and quick to respond, appreciating that the CM^a^ guide was also in recovery, feeling good talking to the CM guide, having access to the CM guide several times per week and after hours, and the CM guide being helpful and motivating the patient.	“I identified with [my CM guide’s] story and a bit about himself. The fact he was able to stay clean for this long and have the job he does. I was impressed by that.” “I thought it was just going to be a very corporate type of meeting, like once a month. But it’s very personalized ... you can be vulnerable ... and it’s a judgment free zone. I wasn’t expecting that level of support from this app.”	12	
	Intervention embedded within a research study	Patients appreciated being able to contribute to research.	“I’ve been addicted for so long, I feel like I have the ability to give back to others. So I was hoping that I’d be able to help other people that are struggling at this time.”	2	
**Barriers (patients)**					
	Testing problems and challenges	Patients expressed frustration with the substance testing process, including difficulty performing the test correctly, the need to have test equipment with you at all times, not having enough time to complete the test, feeling that test results were sometimes inaccurate, running out of test supplies, test kits being too big and bulky, and taking too long to get replacement supplies.	“I took one with my camera, my photo roll, so that was invalid. Another one my saliva didn’t go into all the panels so it was defective.” “If the tests themselves were smaller sized and you could carry them in your backpack or something more portable. Even with the size of these, I need to keep them at home, which means that at work or wherever I am, I have to go home to take the test.”	14	
	Module problems and challenges	Patients found some aspects of the educational modules unhelpful, including difficulty understanding due to typographical errors, feeling like homework, being too complicated, being too long, and not having enough content.	“The questionnaires were fine, but those questionnaires, I don’t know, I could’ve been completely positive I was picking the right selection when you’re reading different learning sessions but maybe ones that are a little easier comprehension -- maybe I’m just not up to par on my education -- some were easier and some were a lot harder.”	5	
	Incentive problems and challenges	Patients expressed negative opinions regarding the incentive structure, including incentives taking too long to be added to the card, the value of incentives being too low, and feeling that debit card restrictions were too limiting.	“I know I’m not going to get much from this reward, and the thought of using ignites me more.”	6	
	Challenges due to psychiatric comorbidity	Patients struggled to use the intervention due to psychiatric comorbidity.	“Ever since I’ve been on Klonopin my short-term memory has been really bad.”	5	
	Technology / remote delivery challenges	Patients struggled to use the intervention or connect remotely, including not liking the remoteness of the intervention, difficulty with onboarding, engagement being limited by the phone being broken, being unsure who to talk to about problems with the intervention, difficulty communicating using the app, difficulty connecting to Zoom, difficulty recording the drug test video, inconsistent Zoom meeting number and timing, reminders being unhelpful (too many, content overbearing, etc), the timer on the phone interrupting filming of the drug test, and the timing of prompts to complete a drug test.	“And the Zoom part, I majorly didn’t like that because you have to start out with Zoom, and I’m sitting there looking at the lady and I can’t hear her, I didn’t like that part.” “I’d wake up at 9 or 10 and it had prompted me at like 8am in East coast time and it had only given me a 2-hour window.” “The only thing I can think of is when I try to reach out for help, it makes me text through the app, and then I get a response back through my iPhone’s text messages, so it’s hard to keep track of what’s been sent and what was responded.”	10	
	Lack of human connection	Patients expressed a lack of human connection, including the CM guide having unhelpful advice or not being a good match, bad timing of calls with CM guides, and the time commitment being greater than expected.	“Basically, I was clear to [my CM guide] with ‘Hey, this approach is not a good one for me and we are not a good match’ and I was hoping that somehow it would work out. But when you disagree with something and participate in these talks and debates after a while it gets frustrating.”	4	
**Suggestions for Improvement (patients)**					
	Drug testing	Patients suggested improving the drug testing experience, including allowing more time to complete tests, allowing patients to request replacement drug test kits through the app, providing test kit supplies more quickly and/or automatically when appropriate, and making drug test kits smaller and easier to use.	“If the tests themselves were smaller sized and you could carry them in your backpack or something more portable ... I need to keep them at home, which means that at work or wherever I am, I have to go home to take the test.”	4	
	Educational modules	Patients suggested improving the educational modules, including adding more modules; making modules shorter, more entertaining, more personalized, and easier to understand; and encouraging patients to start modules early.	“Maybe have more of those CBT modules to educate yourself.” “Maybe I’m just not up to par on my education -- some were easier and some were a lot harder.”	6	
	Incentives	Patients suggested increasing the financial incentives for the drug tests and educational modules.	“For me, the reward should be very substantial in order for me not to use.”	2	
	Improving fit into other treatment services	Patients suggested that the intervention could better integrate with the landscape of other treatment services, including advertising Alcoholics Anonymous and Narcotics Anonymous sooner, helping with transportation to appointments, integrating the intervention with clinic visits, providing education about substances and how they affect the brain, and providing additional therapy resources.	“I think it could integrate better with the addictions clinic here. I know they get copies of everything I’m doing, but they don’t mention it. If there’s more, we heard from [my CM guide], there’s more person-to-person accountability, I think that would be help.”	4	
	Onboarding	Patients suggested that the onboarding process could be improved, including explaining all features of the intervention more completely, providing more detailed instructions on how to use the tests, and offering one-on-one orientation.	“Financial incentives will be the main reason people hook up to the program. But also, we need to explain to them that it’s a big commitment program. It’s not just about getting money on the debit card, but it’s a lot of work and accountability that comes with it.”	4	
	Advertising and outreach	Patients suggested that the intervention be advertised to other patients, including developing an outreach intervention to recruit people who are not engaged with the health care system, emphasizing financial incentives, emphasizing that CM guides are in recovery, using flyers to advertise the intervention, and using positive feedback to advertise the intervention.	“I know that I learned about this through my primary care physician, that’s how I found out about this study. I’d only had a [primary care physician] for about a year before that. A lot of people I was using with definitely weren’t seeing doctors, so I don’t know if there’s any type of outreach program that could talk to the homeless or people on the streets to have something available for them.”	8	

^a^CM: contingency management.

#### Facilitators (Patients)

Patients highlighted a range of perceived benefits, with many participants appreciating the value of testing, educational modules, financial incentives, technological convenience, integration with existing care, and the strong sense of human connection offered by the remote format.

##### The Role of Testing

Most patients found substance testing to be a valuable component of the intervention. The ability to see real-time test results provided immediate feedback, reinforcing their efforts to reduce substance use. Many appreciated the randomness of testing, which added an element of accountability, and the flexible window for completing tests made participation more manageable. Even after using substances, some patients still felt encouraged to complete tests rather than avoid them. Additionally, testing allowed patients to demonstrate compliance to family members and to see what substances were present in their system. Passing a drug test often provided a sense of accomplishment, further motivating engagement with the intervention.

##### Helpfulness of Educational Modules

Many patients found the educational modules to be both engaging and informative. Many enjoyed completing them and felt they learned valuable skills that supported their recovery. The modules provided structured content that reinforced healthy behaviors, making them a meaningful part of the intervention.

##### Impact of Financial Incentives

The financial incentives were widely regarded as a beneficial aspect of the intervention. Patients felt that the incentives were a good reason to stay engaged, had a meaningful impact on their daily lives, and were delivered efficiently. Most agreed that the value of the incentives was sufficient and appreciated that the intervention placed limits on what incentive funds could be used to purchase, ensuring they were spent on appropriate items.

##### Integration Into Existing Treatment Services

Nearly all patients noted that the intervention complemented their existing treatment rather than interfering with or detracting from it. Some saw it as a valuable option in an area where treatment services for methamphetamine use were otherwise lacking. Others found it to be a more acceptable alternative to other treatments (eg, inpatient or residential rehabilitation). Several patients noted that participating in the intervention helped them stay engaged with their other mental health treatment.

##### Benefits of Technology and Remote Delivery

The technological aspect of the intervention was well received by patients. Many appreciated the remote nature of the intervention, which allowed them to engage from the comfort of their own environment while maintaining confidentiality. Several reported that the intervention’s reminders helped them stay on track and that the user-friendliness of the interface made it easy to navigate and complete tasks.

##### Accountability and Overall Intervention Benefit

Patients found the intervention helpful in promoting accountability and supporting recovery. Many reported that it helped them reduce their substance use. The structured support from the intervention was reported as helpful by some for working toward self-improvement.

##### Human Connection in a Remote Setting

Despite being a remote intervention, many patients reported feeling a strong sense of human connection. One participant noted, “It’s very personalized, it’s that third-party observer you can be vulnerable with, and it’s a judgment-free zone ... I wasn’t expecting that level of support from this app.” Many patients appreciated the responsiveness and helpfulness of the intervention staff and found it valuable that their CM guides were also in recovery. Many felt a positive connection with their CM guide, had frequent access to them—including after hours—and found their support to be motivating.

##### Research Study Context

Some patients appreciated the opportunity to contribute to research by participating in the intervention. While not a primary focus of their experience, those who acknowledged this aspect felt that their involvement had a broader impact on improving treatment options for others.

#### Barriers (Patients)

Patients described a variety of challenges to engagement, including difficulties with testing logistics, the design of educational modules, incentive structures, psychiatric comorbidities, technology access, and limits in building supportive human connections.

##### Challenges With Substance Testing

Many patients experienced difficulties with the substance testing process. Some found it challenging to perform the tests correctly, while others questioned the accuracy of the results. The need to carry testing supplies at all times (due to the random timing of testing prompts) was seen as inconvenient by many, and some struggled to complete tests within the allotted time. Running out of supplies and waiting too long for replacements often created further frustration. Additionally, some patients found the test kits too large or bulky to carry easily. The timing of test prompts was also a concern, as it did not always align with their schedules.

##### Limitations of Intervention Modules

Patients expressed mixed opinions about the educational modules, with some finding them too long, complicated, or difficult to understand. Others felt that the modules resembled homework, making them less engaging. At the same time, a few participants thought there were not enough modules to complete over the course of the intervention.

##### Concerns About Financial Incentives

While incentives were generally seen as a benefit, some patients found aspects of the incentive structure frustrating. Delays in funds being added to the debit card were a common complaint, and some felt that the value of the incentives was too low. Others found the spending restrictions on the card too limiting, making it difficult to use the funds as they had hoped.

##### Impact of Psychiatric Comorbidities

Some patients reported struggling to engage with the intervention due to cooccurring psychiatric conditions (eg, memory lapses attributed to psychiatric symptoms or medication side effects). These challenges made it harder for them to consistently participate in testing, complete intervention modules, or attend sessions with CM guides.

##### Difficulties With Remote Access and Technology

Some patients found the remote nature of the intervention to be a barrier. Some struggled with onboarding, while others had difficulty knowing who to contact for help with technical or intervention-related issues. Broken phones or unreliable internet access limited participation for some individuals. Issues with the app, communication difficulties, and trouble connecting to Zoom for videoconference calls during the welcome period further complicated engagement. Many patients had difficulty recording the drug test video due to interruptions from other phone apps, which caused them to lose progress. Others encountered inconsistencies with Zoom meeting numbers and scheduling issues, making it difficult to attend sessions. Some found the reminders to be excessive or overbearing, while others were frustrated by the timing of test prompts, which did not always align with their availability. These technical difficulties made it harder for patients to stay engaged and complete intervention-related tasks.

##### Limits in Making Human Connection

While some patients reported positive interactions with their CM guides, others found the experience less helpful. Some felt that their CM guide was not a good match or that the advice given was not useful. Scheduling was also a challenge, with some participants finding that calls with CM guides were poorly timed or that the overall time commitment was greater than expected.

#### Suggestions for Improvement (Patients)

Patients recommended multiple enhancements for the intervention, such as making testing more convenient, redesigning educational modules, adjusting incentives, improving alignment with other services, offering clearer onboarding, and strengthening outreach efforts.

##### Suggestions for Substance Testing

Patients suggested several ways to improve the substance testing process. Many wanted more time to complete tests to better accommodate their schedules. Some recommended the ability to request replacement test kits directly through the app, while others suggested that supplies be provided more quickly or automatically when needed. Several patients felt that the test kits should be made smaller and more portable to make it easier to complete tests when away from home.

##### Suggestions for Educational Modules

To improve the educational modules, patients suggested adding more content to provide deeper education while also making the modules shorter, more engaging, and easier to understand. Some felt that modules should be more personalized to individual needs, and others recommended encouraging participants to begin working on the modules earlier in the intervention.

##### Suggestions for Incentives

A few patients suggested increasing the financial incentives for demonstrating methamphetamine abstinence and for completing educational modules to further enhance motivation.

##### Improving Fit With Other Treatment Services

Patients offered several suggestions to help integrate the intervention more effectively with other addiction treatment services. Some wanted earlier promotion of Alcoholics Anonymous and Narcotics Anonymous meetings, while others suggested assistance with transportation to treatment appointments. Additional recommendations included integrating the intervention with clinic visits, providing more education about substances and their effects on the brain, and offering additional therapy resources to support recovery.

##### Suggestions for Onboarding

To improve the onboarding process, patients recommended clearer explanations of all intervention features and more detailed instructions on how to use the substance tests. Some felt that onboarding should be conducted one-on-one rather than in a group setting to provide more individualized support.

##### Suggestions for Advertising and Outreach

Patients suggested several ways to expand awareness of the intervention and reach more individuals who could benefit from the intervention. Some recommended developing an outreach intervention specifically targeting people with SUDs who are not currently engaged with the health care system. Others felt that advertising should emphasize key intervention benefits, such as financial incentives and the fact that CM guides were themselves in recovery. Suggestions for outreach strategies included distributing flyers and using positive participant feedback as a promotional tool. One patient reflected on the potential impact of greater outreach, saying, “I know a lot of people who aren’t seeing doctors but could benefit from this intervention. Maybe an outreach intervention could reach those people.”

### Description of Clinician Sample

#### Overview

Of 28 clinicians who were identified as providing substance use or other behavioral health services to study participants, 24 were still employed within the health system at the conclusion of the trial and were invited to participate in research interviews. Of these, 13 clinicians completed semistructured interviews and were included in this study. The professional roles of these participants are shown in Table 3. Just under half were primary care providers, while the remainder were psychiatrists, case managers, or nurse care managers. Just over half worked in primary care clinics, with the remainder working in a specialty addiction clinic. Subsequent sections also describe themes from clinician interviews, which are summarized in Table 4.

**Table 3:** Professional roles and settings for clinician participants who completed qualitative interviews (n=13).

Roles and settings		Value, n (%)
**Professional role**		
	Primary care provider	6 (46)
	Psychiatrist	5 (38)
	Case manager	1 (8)
	Nurse care manager	1 (8)
**Setting**		
	Primary care	8 (62)
	Specialty addiction clinic	5 (38)

**Table 4:** Summary of themes identified by clinicians.

Theme		Summary	Example quote(s)	Clinicians, n	
**Facilitators (clinicians)**					
	Benefit to therapeutic alliance	The intervention benefited the therapeutic alliance between clinician and patient, including engagement with the intervention providing an opportunity to build a therapeutic alliance, a perceived low risk of harm to patients from the intervention, the intervention serving as a source of additional income, patient-reported ease of use of the intervention, and an unexpected benefit to the therapeutic alliance.	“I thought the CM program was really nice and I think it was nice for my patient to be able to come in and process his experience as well and share how he felt about it, if it was working or not.”	11	
	Patient factors (which patients might benefit from the intervention)	Clinicians considered individual-level patient factors when deciding to offer CM^a^, including that treatment should align with patient goals, the impact of environmental stability, perceived interest from patients in using CM, patients being connected to the clinic and attending appointments regularly, and smartphone-based CM as an alternative approach to reach patients who were unwilling or unable to engage with conventional treatments.	“The other thing might be, what does the patient’s environment look like? Are they homeless? Are they in a supportive environment? Are they living on their own? Are they living with multiple people? Is it hard to find space for that [CM intervention] on their own? What are some of those micro interactions? What would that look like in their environment and having that conversation? But that’s probably where I would start.”	7	
	Clinician factors (perception of CM being clinically helpful or effective)	Clinicians wanted to use CM for multiple reasons, including the desire to address the need for treatment of methamphetamine use, understanding the treatment’s value and effectiveness and the need to optimize treatment, patient reports of benefit from CM, and benefit to community and public health.	“I think it did provide some patients with maybe an effective treatment as was designed. So, contingency management was helpful. And for others, even if methamphetamine use persisted, it felt like maybe they were able to reduce their use. And then there are folks who explicitly said that their contact with and their relationship with their [CM guides] was really key. That daily engagement with the app and the prompting and notifications was an opportunity to be reminded of, and maybe even reinforce their own recovery goals, and make that kind of a part of their day-to-day life in the midst of what was often otherwise, a lot of other kind of chaos. There’s a sense of being kind of connected to some kind of support, when they weren’t in clinic or weren’t otherwise engaged in a network that was healthy and supportive of them, if they even had such a thing in their lives.”	11	
	Ease of use	Clinicians described systems-level factors that facilitated the use of CM, including a self-sufficient intervention that did not rely on clinicians, receiving updates from the program, ease of the referral process, availability of technical support and troubleshooting, and more than one modality of treatment.	“So having what seemed like it’d be a self-contained, almost like kind of plug-and-play contingency management intervention as an option that we could offer folks was really, really exciting. And I did wonder if things would be maybe quite as smooth or as effective as advertised, but was still really, really intrigued to try it out.”	12	
**Barriers (clinicians)**					
	Equity issues	Clinicians expressed concerns that the intervention may exacerbate inequities, including complex patient factors, issues of equity, accessibility, and accidentally or inadvertently forgoing access to other treatments due to CM accessibility.	“I think we talked about accessibility. So is this something that the patients are actually going to be able to engage with? But for this study, they had to have easy access to internet for this app. So I mean, even that sometimes is a barrier for folks that don’t have phones. Does it fit well with other interventions that are offered in the clinic?”	8	
	Technology, workflow, and usability	Clinicians were concerned about usability and workflow, including adding to clinician work burden, technological challenges experienced by patients, low clinician familiarity, a lengthy onboarding process for patients, difficulties with enrollment of patients, and technological challenges for clinicians.	“If there had been some additional coaching about what I as a provider could be doing to help build the [patients’] motivation and momentum, and then sustain it as they get onboarded [to the intervention], that may have had an effect. But outside of that, because we kind of treated it as maybe more of a medication or an external referral where you just wash your hands of it and just assume everything’s going to fall into place, it didn’t have any change. So I think that is a missed opportunity that we did not consider needing to pursue.”	10	
	Effectiveness concerns	Some clinicians had concerns about how well CM works in the real world and factors that would negatively impact its effectiveness, including sustainability, effectiveness, cost-effectiveness, and quality of life; a perceived lack of impact on patient methamphetamine use; a lack of clarity about the appropriate length of the intervention or how to handle repeat intervention episodes; patients being falsely reassured; the incentive structure; variability of experiences with CM guides; and perceived low engagement of patients.	“Well, I think ... is it effective is probably the first thing we should consider. Does it help patients? And then is it cost effective? What’s the cost? And especially what’s the cost to the patient? Because many of our patients don’t have high incomes and couldn’t take on extra costs for care.”	12	
	Privacy and monitoring concerns	There were concerns related to privacy, surveillance, and monitoring of people who use drugs, including psychosocial treatment being offered or controlled by a company outside of the clinic’s health care system and patients not wanting to participate in research.	“Very, very broadly, from a 30,000-feet view. I worry about the level at which people who use drugs are surveilled. Because I think there was a video component where people swab and I just worry about that, ongoing surveillance of people who use drugs.”	3	
	Integration with other clinical services	Clinicians felt that CM could have been better integrated into the clinical work setting, citing limited capacity to integrate new workflows, disconnect or lack of alignment of the CM intervention with in-clinic visits, limited clinician involvement with the intervention, and perceived clinician disconnectedness.	“Building out a new kind of intervention and clinical offering like this always takes a lot of time. And doing a research project where people are going to have three months of active intervention and the whole thing may last a year and a half or so, that just may not be enough time for it to really take hold and become fully integrated into the clinic’s way of thinking and doing things. And it’s hard. And with anything new, I mean it’s obviously hard to do that in general. It takes a lot of coaching, and meetings, and so forth, and that is just hard to do. The other thing is there’s lots of interventions, and providers’ focus is pulled in lots of different directions, and so that also makes building something like this difficult.”	11	
**Suggestions for improvement (clinicians)**					
	Education	Clinicians suggested focusing on education when implementing CM, including establishing clear rules and expectations for patients who participate, promoting clinician buy-in by showing data on the need for such interventions, educating clinicians about the nature of the intervention, creating a patient-specific pamphlet, and incorporating the intervention into medical education.	“I think any buy-in from the provider side is extremely important. And so, understanding how many patients that you see on a daily basis actually do have issues with stimulant use disorder or substance use disorder in general. That is kind of a question that when asked is generally a little surprising. And I think that helps to facilitate buy-in to say, ‘Yeah, this is an issue that we need to have more resources or ways to address, and maybe I’m not doing enough to understand the community that I’m serving and their need for that resource.’”	6	
	Engagement	Clinicians suggested improving engagement from patients and clinicians by designing the intervention to encourage person-to-person interactions and social connections and keeping clinicians more engaged with the intervention and informed about the intervention rollout.	“Some of the things that I really like for studies like this would be to get an update on how the project’s going. So a newsletter is kind of cool to get once in a while, which could be in the form of an email that just says, ‘Here’s the progress, don’t forget about us. These are the key things. Here’s a link to a website with more details. Here’s who you should contact.’ Things like that. And that is coming monthly or quarterly, depending on recruitment.”	2	
	Collaboration using existing resources	Clinicians suggested using existing resources to improve collaboration when implementing CM, including integrating the intervention into existing clinical workflows, having an intervention superuser in the clinic, collaborating and using existing support, and obtaining input from relevant stakeholders.	“If you’re working in a neighborhood clinic with maybe primarily Amazon employees, that’s going to be a different group with different needs than someone that who, ‘The average patient,’ whatever that looks like at [county safety net mental health center]. And I guess what would be good is, part of implementation would be getting some input and buy-in from clinicians and staff, and even administrators locally. And if there are opportunities to get input from patient advisory boards, or members of governing boards that have, or folks with lived experience, that would actually be super awesome. And we didn’t have as much opportunity. We tried to involve some of our peer specialists early on, but doing that more vigorously would be helpful.”	11	
	Alignment with existing clinical structure	Clinicians felt that the intervention could be better aligned with other clinical structures and practices, including leveraging or synergizing patients’ experiences with the CM intervention in coordination with other clinical interventions.	“I think something that would’ve helped me be aware and reminded me of it would’ve been useful. Ideally a patient coming in and having it somehow be associated with the visit. If there was a patient on my schedule with methamphetamine history or with current methamphetamine use as a diagnosis and saying, ‘Hey, this person is eligible, potentially don’t forget about meth use ...’ That would’ve been great. And that’s where the way we’re working toward with clinical care, which those are the workflows that fit into with research stuff, is to tie in with whatever decision support on treatment options that really integrate it into those workflows. That would’ve been the most useful for me.”	3	
	Reward more outcomes that don’t require abstinence	Clinicians expressed a desire for the intervention to be more thoughtful about incentivizing outcomes that are not inherently abstinence-based.	“When you look into the contingency management literature, at least what I see, it’s heavily focused on abstinence. And the people who are participating are people who say, ‘I want abstinence.’ But even in our patient population, when people are talking about abstinence, it comes and goes. Even with my one patient who I got into the [CM intervention], he goes back and forth on that a lot. Sometimes he wants abstinence ... I don’t know, I’m curious about contingency management being done in a harm- reduction model more.”	2	
	Develop infrastructures	Clinicians suggested developing infrastructure to support programmatic implementation, including promoting more holistic treatment, strengthening workforce engagement, supporting targeted patient population or panel management, and identifying effectiveness markers.	“Do we have a population that would benefit from it? And I think in this urban setting, absolutely. Do we have the engagement of the majority of providers? Are we going to put it out there and have providers that are willing to engage patients and attempt to use the program? And then the capture rate, do we have enough patients? Can we get to enough patients to start to work up towards that success rate, and how are we to measure that? And the resources just to be issuing phones and incentives, and making it reasonably accommodating for a patient to show up, give test samples, things like that. How can we work through some barriers that may make a person less likely to continue? Because once they miss one session, it’s easier to miss a second and a third. So reducing barriers on the clinic side, the patient’s side, and do we just have the resources to continue that?”	8	

^a^CM: contingency management.

#### Facilitators (Clinicians)

Several positive themes emerged from the clinician interviews, with most clinicians endorsing a perception that CM is an effective treatment, believing that the intervention would be helpful for patients with certain characteristics, and expressing a positive view of the intervention operating as a self-contained program.

##### Benefit to Therapeutic Alliance

Most clinicians expressed positive sentiments toward CM as an opportunity to build greater therapeutic targets/alliance via a modality with low perceived risk of harm to patients that could also generate additional income for patients.

##### Interest in Patient Selection/Optimization

Most clinicians appreciated CM as a potentially effective treatment that is in alignment with patient goals and expressed particular interest in optimizing patient selection. They reported that patients with relatively intact living environments and those who demonstrated upfront interest in the intervention may be most receptive and most likely to benefit from smartphone-based CM. They further noted that for some patients who were unwilling or unable to engage with conventional treatments, mHealth represented a potentially attractive alternative therapeutic modality.

##### Perception of CM Being Effective

Clinicians appreciated having CM as a treatment option when no other evidence-based interventions were available. Several clinicians appreciated that CM was evidence-based and noted that patients reported benefiting from it. Some clinicians proposed that the impact of CM may extend to benefit the patient’s greater community.

##### Ease of Use

The intervention was perceived by many clinicians to be self-sufficient, and they felt it did not result in increased clinical workloads. Many appreciated the updates they received from the intervention and the ease of the referral process. Many also expressed positive sentiments about the availability of technical support.

#### Barriers (Clinicians)

Perceived barriers to CM uptake also emerged from the clinician interviews, with several clinicians identifying equity, accessibility, usability, and workflow concerns.

##### Equity and Accessibility

Some clinicians discussed equity-related concerns—for example, that clinicians might preferentially offer CM to patients who engage more frequently with in-person treatments. Similarly, clinicians expressed concerns about inadvertently not offering other non-CM treatments due to the perceived accessibility of CM. Several clinicians also expressed worry that a lack of access to mobile phones could create inequities in who has access to smartphone-based interventions. Several clinicians described the difficulties of enrolling or engaging patients in this clinical population, noting that complex and challenging psychosocial factors may impede engagement. For example, 1 clinician stated, “We saw, a lot of the patients that we had, had multiple cooccurring issues ... chronic diseases, medical issues, multiple substance use, mental health diagnoses ... which definitely makes for a very challenging population to reach.”

##### Usability

Clinicians expressed several concerns about usability issues hindering patient engagement. For example, some clinicians were concerned that patients could have problems navigating the smartphone app. One stated, “I think my patient had trouble uploading videos and kind of got frustrated and gave up.” Similarly, some clinicians also expressed not being familiar with how to use available tools for tracking their patients’ progress with the intervention. Several clinicians also described concerns that it took too long for patients to complete onboarding. One clinician stated, “On the sort of less helpful side, obviously, some [patients] really struggled to get in, and remain engaged in the intervention. The long timeline was really frustrating, and didn’t allow us to strike while the iron was hot.” Other clinicians agreed that the enrollment process of getting their patients into the CM intervention was too complicated and required additional effort and time from the clinician.

##### Effectiveness

Clinicians were particularly concerned about the effectiveness of the intervention for specific patient populations, especially those with comorbid serious mental illness. Several expressed concerns about the lack of evidence for the appropriate length of CM. Others agreed that they would not know how to approach the issue of repeating treatment for patients who require multiple courses of CM, including how costs and financial incentives would be handled for these patients. Clinicians raised a number of concerns related to sustainability as well. They questioned how to support their patients’ recovery goals once treatment ended, including one who stated, “One of the things that I really feel guilty about not spending more time thinking about with individual patients is that regardless of the duration of treatment, treatment comes to an end. And with contingency management, cessation of treatment, it’s associated with return to use at higher levels than it would be for others like CBT for substance use disorders, because contingency management isn’t skill-based.” Furthermore, clinicians were concerned that their patients might be misinformed or falsely reassured that CM alone (without other interventions) would be sufficient for treating their methamphetamine use, potentially leading some patients to forego other treatment options. Another challenge that clinicians noted was the variability of patients’ experiences with CM coaches, and hearing from patients who perceived their CM coach to be unsupportive. A few clinicians also thought the financial incentives were not adequate. Additionally, several clinicians highlighted the delay in the treatment effect of CM when compared to some pharmacologic interventions, which led to decreased interest and motivation. For example, one stated, “In what ways was it not helpful? Getting excited about a treatment or excitement is probably not the right word, but when you’re talking with people and talking through treatment options and then agreeing on a specific treatment, it’s helpful to move on that quickly and to start things going in that direction as soon as possible. And that’s a huge reason why everybody likes medications because you can start it today, you can start doing something today, and it’s probably a reason why some people get disappointed with therapy because it could be months out that you actually start to notice anything.”

##### Ethics

Clinicians noted a few ethical dilemmas that were barriers to implementing the CM intervention. Some expressed concerns about intrusion into patient privacy, given that patients recorded themselves when conducting saliva-based drug tests. Others noted the difficulties of getting patients who use methamphetamine to engage with a smartphone-based CM intervention, due to potential concerns of their substance use being monitored. Similarly, another clinician expressed negative sentiments toward the intervention being offered by a private company outside of the clinic’s health care system.

##### Integration With Other Clinical Services

Clinicians raised a number of organizational and capacity issues that would prevent the successful integration of smartphone-based CM into their clinical practice. Many clinicians expressed worry that integrating smartphone-based CM in their clinics in a more sustainable manner (ie, beyond the scope of this small pilot study) could add to their already heavy clinical workloads. Some felt that the intervention’s content and timeline could have been better aligned with the timing of their patients’ clinical visits. Some were additionally concerned about the fit of the intervention content with other interventions they used. While many clinicians enjoyed having a stand-alone CM intervention, some also felt disconnected and wanted to have a better understanding of their patients’ experiences with it. Consequently, a few clinicians reported they were not very engaged with the intervention, which hampered their ability to support patients’ involvement with it.

#### Suggestions for Improvement (Clinicians)

In addition to the facilitators and barriers identified by the clinicians in our study, they expressed specific recommendations that could enhance the clinical delivery and implementation of smartphone-based CM.

##### Education and Engagement

Clinicians emphasized the importance of having education and training for all clinical staff to collaborate effectively when integrating the mHealth CM intervention into their clinics. Clinicians also described the importance of having buy-in from all staff members. Another suggested creating more opportunities for interpersonal interactions and social connections in the clinic as a way to increase clinician engagement and also improve future implementation.

##### Collaboration and Alignment With the Existing Clinical Environment

Clinicians suggested that mHealth CM interventions should provide robust technological support, limit the number of administrative tasks, and continue to optimize usability for clinicians, ancillary staff, and patients. Additionally, they expressed interest in having a CM intervention that is better aligned with their existing clinical structure and visits.

##### Non–Abstinence-Based Outcomes

Many clinicians expressed a desire for the intervention to be tailored to the needs of their patient populations and clinical approaches, which often emphasized harm reduction rather than abstinence.

##### Development of Clinical Infrastructure

In addition to ensuring that there is appropriate infrastructure to support a CM intervention in the clinical environment, clinicians also expressed the need to have feasible “effectiveness markers” to help monitor and track their patients’ progress with the intervention. Finally, they felt that the initial duration of the CM intervention should be more individualized and discussed in advance with patients, leaving open the possibility of repeat enrollment when appropriate, in order to ease transitioning from CM to other treatment modalities.

## Discussion

### Principal Findings

This study provides important insights into patient and clinician experiences and perspectives related to a smartphone-based CM intervention that was offered to patients who use methamphetamine. Together, these findings enhance our understanding of the opportunities and limitations associated with smartphone-based CM models and highlight practical recommendations for the implementation of such interventions in clinical settings.

Across both patient and clinician groups, there was a shared recognition that CM fills a critical treatment gap, especially due to the lack of widely available evidence-based interventions for patients who use methamphetamine. Both groups perceived the financial incentives offered by the CM intervention to be a valuable and safe strategy to increase motivation for engagement and recovery. Patients found the educational modules useful and often found the CM guides to be helpful. Clinicians viewed CM as a research-supported treatment that provided an opportunity to strengthen their therapeutic alliance with patients and meaningfully engage those who might otherwise not respond to other available approaches.

Technological challenges emerged as a common barrier for both groups. Patients reported difficulties with video-based substance testing, app navigation, and access to reliable internet service. Clinicians expressed concerns about potential technology-related barriers, especially for patients with more profound social and socioeconomic barriers. These underscore the importance of designing CM platforms that are accessible, easy to use, and responsive to the needs of individuals with varying levels of digital literacy.

There were also some notable differences in the scope of concerns raised by each group. Patients tended to focus more on the immediate, concrete features of the intervention, such as the helpfulness of the educational modules, the timeliness of financial incentives, and the perceived support from CM coaches. In contrast, clinician responses were more focused on broader systems-level factors, raising issues related to equity, clinical integration, long-term sustainability, and ethical use of the intervention. Clinicians highlighted concerns about how factors related to socioeconomic status could impact which patients they select to offer CM. They also questioned the traditional abstinence-based model of CM, including its long-term impact on recovery, its alignment with other ongoing treatments, and whether a non–skill-based approach could sustain benefits once the intervention ended.

These findings highlight relational and systemic factors underlying patient and clinician perspectives. Patients’ emphasis on perceived support from CM coaches aligns with clinicians’ discussion of the intervention’s potential to strengthen therapeutic alliance [12]. Both groups recognized that better integration with existing clinical services could improve engagement and outcomes, emphasizing that the intervention’s effectiveness is enhanced when it is embedded within broader care and when patients feel connected to supportive clinicians. Clinicians’ focus on equity and sustainability further underscores systemic challenges, including resource allocation, staffing, and broader social determinants of health [5,6].

Moreover, our findings extend existing research on digital CM by explicitly examining both patient and clinician perspectives. While prior studies have quantitatively evaluated smartphone-based CM for various substance use populations [7-11], few qualitative studies have explored the experiences of both patients and providers concurrently, and none have focused specifically on methamphetamine use [12]. This dual perspective allows identification of points of convergence and divergence, such as shared recognition of CM’s motivational benefits and technology-related barriers, as well as differences in how immediate versus systemic issues are prioritized. Recognizing these complementary perspectives can inform the design of future digital CM interventions, emphasizing both user-centered usability and system-level feasibility.

These results must be interpreted within the broader context of health care delivery during the COVID-19 pandemic. Clinics that participated in this study were dealing with high clinical demand, staffing shortages, and other COVID-19–related clinical care adjustments. The CM intervention used in the pilot study was designed to be fully remote to minimize potential additional burdens to an already strained system. As a result, clinician involvement in and engagement with the intervention were limited.

Social determinants of health (eg, poverty, housing instability, and limited access to technology) further shaped patients’ experiences. Some individuals struggled with remote onboarding, video-based testing, and the use of mobile apps. These challenges were even more difficult among patients with cooccurring psychiatric conditions or unstable living environments. These challenges underscore the importance of designing CM interventions that are both flexible and responsive to real-world barriers.

Unlike prior studies on CM, which often focused on other substances or used more integrated in-person models (eg, onsite onboarding or supervised substance testing), this CM intervention was fully remote and targeted methamphetamine use. While remote delivery increased accessibility for some patients, it also introduced unique challenges related to patient engagement, technology use, and delayed reinforcement. Compared to in-person CM, which often provides more immediate and structured rewards, the digital format may have resulted in lower motivation for some patients.

### Clinical Implications and Recommendations

Based on these findings, several practical recommendations can guide future implementation of smartphone-based CM interventions.

First, greater integration with existing clinical services may help bridge the gap between stand-alone digital tools and routine clinical care. Clinics can use specific operational steps, such as providing in-person onboarding, completing a brief app demonstration, and ensuring that patients are able to successfully submit a test video before independent use. Identifying a clinic point-person to monitor CM progress and embedding CM-related reminders into the electronic health record can further support workflow integration and sustainability.

Second, CM interventions may benefit from a broader set of rewardable behaviors, beyond strict abstinence from methamphetamine (or other substances). Incorporating financial rewards for harm reduction outcomes, such as reduced use, therapy attendance, or other prorecovery behaviors that do not require abstinence, could support individuals who are at various phases of their recovery journey or who have difficulty with abstinence. Additionally, rewarding the completion of substance tests, regardless of result, may help improve engagement with substance testing among all patients. These strategies align with clinical approaches that emphasize reinforcing small, achievable steps to promote sustained behavior change.

Third, app-specific factors should be considered to optimize user experience. More immediate access to rewards, larger and more celebratory notifications for negative test results, and stronger integration with support networks (eg, involving family or referring clinicians) could boost patient motivation to engage with the intervention and reduce methamphetamine use. Offering portable “travel testing kits” may be helpful for some individuals who would otherwise miss tests due to the inconvenience of keeping supplies on hand.

Finally, continued attention to equity and digital accessibility is essential. Ensuring that patients have reliable access to technology, minimizing onboarding complexity, and providing personalized support when needed will be critical for the success of smartphone-based CM interventions in real-world settings.

### Limitations

Several limitations should be considered when interpreting the findings of this study. The original pilot study was conducted within a single multisite academic health system and included patient participants with high rates of cooccurring substance use and mental health conditions. As a result, the sample represents a population with complex treatment needs, and the findings may not be generalizable to other clinical settings or patient populations. Patient participants included those who completed initial onboarding steps required to be exposed to the smartphone-based CM intervention, and thus the sample does not include perspectives of patients who were not capable of completing those initial onboarding steps. The clinician participants often had little direct involvement with the CM intervention, and their perspectives may have been primarily influenced by information they heard from their patients or from periodic updates about their patients. Some clinicians provided input regarding how the intervention should be modified and offered to patients for the pilot, which may have also influenced how they responded to some questions during the qualitative interviews. The sample did not include clinicians who may have lacked the interest or time required to participate in research. The sample size used in this formative research was based primarily on the number of patients and clinicians who were eligible and willing to participate during the study period rather than a sample size designed to ensure thematic saturation; thus, it is possible that additional benefits, drawbacks, and ideas for improvement could emerge with future studies that use larger or more diverse samples.

While qualitative methods provide rich insights into participant experiences, they are inherently subjective and rely on self-reported data, which can be influenced by recall bias or social desirability. Future studies could address these limitations by integrating multimodal data collection, combining qualitative interviews with quantitative measures such as app usage logs, substance use outcomes, and clinician-reported outcomes, to supplement and validate self-reported experiences and to better capture the benefits, drawbacks, and opportunities for improvement of smartphone-based CM interventions. Recruiting a broader and more diverse sample of both patients and clinicians, including those who face challenges completing onboarding or who may be less engaged with research, could improve generalizability and capture a wider range of perspectives. Additionally, longitudinal follow-up or repeated interviews may help reduce recall bias and capture evolving experiences with the interventions over time. The version of the intervention that was tested in the pilot study was tailored by the research team in an effort to focus specifically on methamphetamine use and to improve fit with the clinical settings where it was used; therefore, the experiences of participants in this study may not reflect experiences that others may have with the same digital health program if it was focused on use of other substances or customized for other settings.

### Conclusions

Smartphone-based CM interventions represent a promising approach to addressing methamphetamine use, particularly in settings where traditional in-person CM is difficult to implement. While both patient and clinician groups reported clear benefits of remotely delivered CM, successful use in clinical practice will require integration with existing services, simplified onboarding, and supports that address equity and digital accessibility.

At a policy level, our findings highlight the importance of establishing reimbursement pathways, promoting digital equity, and developing guidance for expanding CM for broader recovery-oriented outcomes.

Future studies should evaluate hybrid delivery models, test broader rewardable behaviors, incorporate objective and longitudinal data, and recruit more diverse patient and clinician samples. These efforts will help clarify how smartphone-based CM can be optimized to meet the needs of patients and health systems in real-world settings.

## Appendix

Multimedia Appendix 1 Guide used to interview patients.

Multimedia Appendix 2 Guide used to interview clinicians.

## Abbreviations

CBT: cognitive behavioral therapy
CM: contingency management
mHealth: mobile health
MUD: methamphetamine use disorder
OUD: opioid use disorder
SUD: substance use disorder

## Data Availability

The datasets generated or analyzed during this study are not publicly available due to restrictions in the informed consent provided by participants. Deidentified data may be made available from the corresponding author upon reasonable request.
