# Supplementary material for: Smartphone-Based Contingency Management for Patients Who Use Methamphetamine: Qualitative Analysis of Patient and Clinician Perspectives
Source: JMIR Form Res. 2026 Feb 19;10:e80808. doi: 10.2196/80808 (PMC12963966; doi:10.2196/80808)
Supplement: Multimedia Appendix 1 [file formative_v10i1e80808_app1.docx]

**Interviewer Prompt:**

- We are hoping to learn more about your experiences with the DynamiCare Program.
- Your feedback is appreciated and will help us understand if the DynamiCare Program could be useful to other individuals in the future.
- There are no right or wrong answers, and you are welcome to skip any questions. Your answers will be kept confidential, and will only be used for research purposes, unless we believe you are at risk of hurting yourself or others.
- Do you have any questions or concerns before we begin?
- Great! Please keep in mind that for the questions below, I’ll be asking about your experience with the FULL DynamiCare Program, including your experiences with the recovery coach, substance testing and the rewards, and the smartphone app.

**Interview Questions**

**DynamiCare/Technology**

1. What were some reasons you decided to try the DynamiCare program?
2. What parts of the program did you find most helpful, and why? This could include the recovery coaching, substance tests, financial rewards, self-guided therapy modules, or any other aspects of the program.
3. What parts of the program did you not find helpful?
4. Was there anything that made the program difficult to use?
5. What do you think could help make the program better or easier to use?
6. We know folks can have many reasons for not being able to complete their substance tests when they receive the prompt. Can you share some reasons you didn’t complete the substance tests when your phone alerted you?
7. Can you share your thoughts on the value of the rewards (for the substance tests the CBT modules, and the recovery coaching)?

**Opioid and Methamphetamine Use**

1. In what ways did the DynamiCare program help you manage your substance use?

**Contingency Management Support**

1. In what ways did the DynamiCare program impact (add to or subtract from) other treatments you are getting or programs you have been involved with? This could include counseling or case management.
2. What other support or resources would be helpful while using this program?
3. Do you have any suggestions for ways that we could introduce this program to other patients?

**Miscellaneous Questions**

1. What aspects of the program have worked in the way you expected them to? What aspects of the program worked in a way that you didn’t expect?
2. Is there anything else you think we should know about your experiences using DynamiCare?
3. We spoke about this at the beginning of the study, but I’d like to confirm with you again. Once we have completely finished the study, we can contact you by email to share the results of the study with you. Is that something you’d like us to do?

YES NO
