# Supplementary material for: Smartphone-Based Contingency Management for Patients Who Use Methamphetamine: Qualitative Analysis of Patient and Clinician Perspectives
Source: JMIR Form Res. 2026 Feb 19;10:e80808. doi: 10.2196/80808 (PMC12963966; doi:10.2196/80808)
Supplement: Multimedia Appendix 2 [file formative_v10i1e80808_app2.docx]

**Clinician Interview Guide**

**Last Updated June 7, 2021 (Version 1.1)**

**Introductions**

- Researcher will introduce themselves, state their role at the University of Washington, and state their role in the study.
- Invite interviewee(s) to introduce themselves if they are not already known.

**Purpose of this interview**

- We’re trying to understand clinicians’ experiences with the DynamiCare Study so we can learn what was helpful, what worked for clinicians, and what didn’t work so well.
- Our goal is to learn from the experience and improve future programs like it.

**Audio recording**

- What you say here is **confidential**.
- We are asking clinicians if they’re willing to be **audio recorded** (if in person) or **video recorded** (if Zoom). We only do this so we can retain accurate information. We will never identify you as being a participant in this research.
- It’s not a problem if you do not wish to be recorded.
- Do you have any questions about recording?
- Would it be okay with you if we (audio/video) recorded this interview?

**Consent Form**

- I have emailed you an informed consent form, which I’ll ask you to review. You can ask any questions about it before we start the interview.
- (Ensure clinician gets the email. Encourage them to review the form.)
- Before I move on, I want to pause here and take a few minutes to answer any questions that you have.

**Start the (audio/video) recording, if clinician agreed.**

- I’m starting the recording now.
- Could you please verbally indicate your consent to being recorded?
  - Verbal consent to being recorded must be included in the audio recording.

**If done in a group format:**

I’d like to quickly review a few ground rules for our discussion today:

- Please be respectful of others’ opinions even if you don’t agree.
- Please try to stay on topic, we may need to interrupt so we can cover all the material.
- Be aware of your participation.
- Keep focus group discussion confidential.
- *What other ground rules would you like to add?*

**Interview Questions**

**Initial Expectations/Concerns**

1. What were your initial expectations and concerns about using a Contingency Management program like DynamiCare?
2. What factors do you consider when deciding whether to introduce a program like DynamiCare to a patient?

**Clinical Workflow/Implementation**

1. In thinking about your involvement with this intervention – what worked well for you and what didn’t work well?
   1. Were there parts of the study, or of DynamiCare, that fit well into your clinical workflow, or parts that did not fit well?
2. What could have made this program easier or more feasible for you to work with?
3. What are some potential problems that you could envision if a program like DynamiCare were to be used ongoing in your practice?

**Patient care / Substance use treatment**

1. In your opinion, how did the DynamiCare program help patients? In what ways was it *not* helpful?
2. How has this program changed the way you deliver substance use treatment-related patient care?

**Miscellaneous questions**

1. What factors are important to consider when deciding whether a new intervention, like DynamiCare (a Contingency Management, smartphone-based intervention) should be implemented clinic wide?
   1. **Follow up / clarification:** More specifically, what kinds of barriers or challenges exist? What facilitators would be important to ensure smooth/sustainable clinic-wide implementation?
